# 误诊为慢性淋巴细胞白血病的伴TP53基因突变的惰性白血病性非淋巴结型套细胞淋巴瘤1例报告并文献复习

**DOI:** 10.3760/cma.j.cn121090-20241205-00538

**Published:** 2025-02

**Authors:** 彤璐 邱, 亚平 张, 琰 王, 雨洁 吴, 文瑜 施, 奕 夏

**Affiliations:** 1 南京医科大学第一附属医院，江苏省人民医院血液科、淋巴瘤中心，南京医科大学血液研究重点实验室，江苏省肿瘤个体化医学协同创新中心，南京 210029 Department of Hematology, Lymphoma Center, Key Laboratory of Nanjing Medical University, the First Affiliated Hospital of Nanjing Medical University, Jiangsu Province Hospital, Collaborative Innovation Center for Cancer Personalized Medicine, Nanjing 210029, China; 2 南通大学附属医院血液科，南通 226001 Department of Hematology of Affiliated Hospital of Nantong University, Nantong 226001, China; 3 南通大学附属医院肿瘤科，南通 226001 Department of Oncology of Affiliated Hospital of Nantong University, Nantong 226001, China

## Abstract

白血病性非淋巴结型套细胞淋巴瘤（nnMCL）患者临床上通常表现为惰性病程，当无症状及治疗指征时，可采取观察随访。本文对1例被误诊为慢性淋巴细胞白血病（CLL）且携带TP53基因突变的nnMCL患者进行了回顾性总结，并结合文献复习。此病例强调了nnMCL与CLL鉴别诊断的重要性，且对于nnMCL患者，即使其存在TP53基因突变等高危生物学标志，采取严格的观察等待策略可能比立即进行治疗更适宜。

## Abstract (en)

Patients with leukemic non-nodal mantle cell lymphoma（nnMCL）typically exhibit an indolent clinical course, and when asymptomatic and without treatment indications, a watchful observation follow-up can be adopted. This article presents a retrospective summary of a case of nnMCL, misdiagnosed as chronic lymphocytic leukemia（CLL）and carrying a TP53 mutation, along with a literature review. The case highlights the importance of differential diagnosis between nnMCL and CLL, and suggests that for nnMCL patients, the presence of high-risk biological markers such as TP53 mutations does not necessarily indicate an immediate need for treatment; rather, a strict watch-and-wait strategy may be a more appropriate option.

套细胞淋巴瘤（mantle cell lymphoma，MCL）是一种起源于成熟B细胞的非霍奇金淋巴瘤。MCL临床表现具有异质性：常见的经典型MCL（cMCL）呈侵袭性病程，疾病进展迅速，而相对少见的白血病性非淋巴结型MCL（leukemic nonnodal MCL，nnMCL）常为惰性起病，临床上需与慢性淋巴细胞白血病（chronic lymphocytic leukemia，CLL）鉴别[1]。MCL的遗传不稳定性极高，分子遗传学上的特征性表现是t（11;14）（q12;q23）并导致cyclin D1过表达，继而MCL细胞发生大量影响多个通路的基因突变[2]。TP53基因突变及缺失是其中关键的改变，其与较短总生存（OS）期及无进展生存（PFS）期相关[3]，是极高危MCL的标志性改变。nnMCL在获得TP53基因突变和（或）缺失后可能进展为高度侵袭性MCL[4]。但也有研究表明，相较于伴TP53基因突变的cMCL患者，伴TP53基因突变的nnMCL患者有较高的5年生存率（48％对17％）[5]。现对我院1例诊断为伴TP53基因突变的nnMCL患者的临床资料进行整理并文献复习。

## 病例资料

患者，男，68岁。2008年体检示WBC 10.3×10^9^/L、淋巴细胞计数5.4×10^9^/L、HGB 147 g/L、PLT 269×10^9^/L，未予重视。2012年体检发现WBC 11.05×10^9^L、淋巴细胞计数6.7×10^9^L、HGB 159 g/L、PLT 213×10^9^L。患者于当地医院就诊行外周血流式细胞术肿瘤细胞表型示：淋巴细胞占有核细胞比例为62.0％，其中B淋巴细胞占淋巴细胞比例为54.00％，主要表达HLA-DR、CD5、CD11c、CD19、CD20、CD22、CD23、CD25，CD5^+^CD19^+^CD23^+^细胞约占29.69％。外院诊断为CLL且暂无治疗指征，定期复查血常规（表1）及浅表淋巴结超声，门诊随诊。2015年7月15日血常规示WBC 25.1×10^9^L，外院予口服苯丁酸氮芥（无治疗指征），患者服用至2017年5月17日。服药期间患者PLT及HGB正常。2017年5月17日，患者于我院就诊，查体浅表淋巴结未及肿大，肝脾肋下不可及。完善外周血流式细胞术肿瘤细胞表型检查（图1）。二代测序（NGS）示：CCND1基因突变检测阳性（p.C47S；突变比例9.32％）；TP53基因突变检测阳性（p.H178D；突变比例18.16％）；KMT2D基因突变检测阳性（p.G4272D；突变比例52.78％）。FISH示：无TP53基因、IGH-CCND1融合基因阳性。外周血染色体核型（CpG+IL-2刺激）示：46,XY,t（11;14）（q13;q23）[6]/47,XY,idem,+15[1]/48,XY,+4,+15[1]/46,XY[2]。免疫球蛋白重链可变区（IGHV）基因有突变，IGHV 1-8*01，符合率为96.88％。患者诊断为nnMCL，简化套细胞淋巴瘤国际预后指数（sMIPI）：4分、中危；联合Ki-67的套细胞淋巴瘤国际预后指数（MIPI-c）：低中危。评估患者无症状，暂无治疗指征，停服苯丁酸氮芥。后患者规律定期门诊复诊。2017年7月外院行右侧腋窝淋巴结穿刺，穿刺标本免疫组化示：CD3（−）、CD20（+）、CD79a（+）、CD5（+）、cyclinD1（+）、CD10（−）、Bcl-6（−）、MUM-1（−）、Ki-67（10％+）、CD43（−）、Bcl-2（+）、CD21（+）、CD23（+）。患者2018至2023年均来院随诊，病情持续稳定（表2）。2023年9月颈部+胸部+全腹部增强CT示：两侧颈动脉间隙旁、颈后部、两侧锁骨上区及两侧颌下见小淋巴结显示，增强后可见轻度强化，较大者短径6 mm；纵隔淋巴结显示，大者短径约9 mm，两肺门未见明显肿大淋巴结影；脾偏大，两侧腹股沟区小淋巴结显示，腹盆腔、腹膜后区未见肿块或肿大淋巴结影。截至2024年10月患者未启动治疗，期间淋巴细胞计数、HGB、PLT变化见表1。2024年10月复查外周血流式细胞术肿瘤细胞表型示：异常单克隆B细胞占有核细胞比例为58.9％，肿瘤细胞表达CD5、CD22、CD79b、CD20，弱表达CD23、CD200、Kappa，不表达FMC7。外周血细胞形态学检查显示淋巴细胞体积较小，形态规则。

**表1:** 伴TP53基因突变的白血病性非淋巴结型套细胞淋巴瘤患者2008年至2024年血常规结果

检验日期	WBC（×10^9^/L）	淋巴细胞计数（×10^9^/L）	HGB（g/L）	PLT（×10^9^/L）
2008年8月	10.3	5.4	147	269
2011年8月	14.7	7.2	159	265
2012年3月	11.0	6.7	159	213
2013年4月	17.3	11.4	146	255
2014年1月	15.2	7.8	137	282
2016年10月	14.4	9.7	153	216
2017年11月	15.1	11.3	147	229
2019年6月	13.6	25.9	154	212
2020年7月	21.6	15.4	147	219
2021年4月	20.2	15.3	157	178
2022年4月	17.3	12.8	166	212
2023年5月	15.0	10.9	150	185
2023年9月	14.4	9.0	143	186
2024年3月	11.7	7.4	142	175
2024年9月	14.3	9.8	145	170

**图1:**
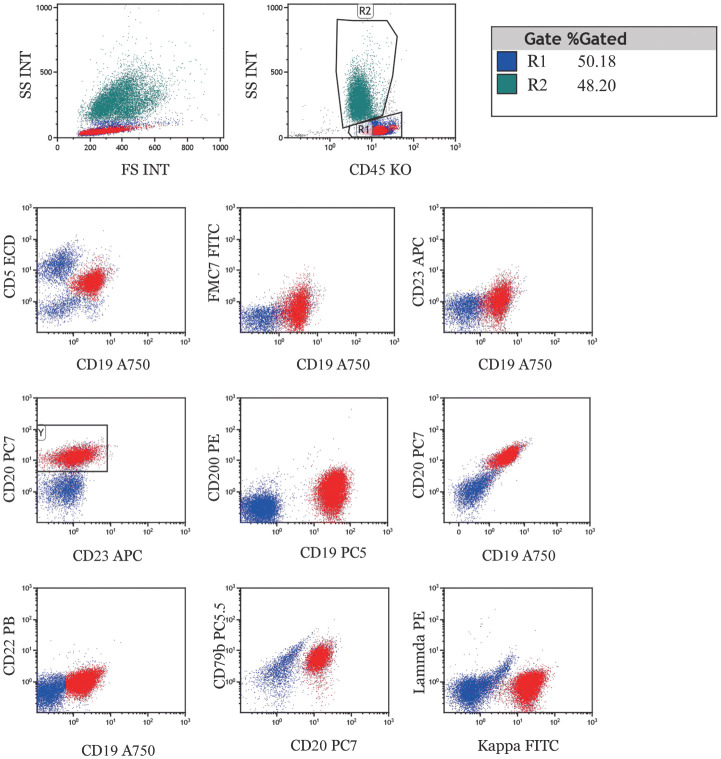
伴TP53基因突变的白血病性非淋巴结型套细胞淋巴瘤患者2017年外周血流式细胞术肿瘤细胞免疫表型 **注** 异常单克隆B细胞占有核细胞比例：30.12％，肿瘤细胞表达CD5、CD79b、Kappa、CD20，弱表达CD22、CD23、CD200，不表达FMC7

**表2:** 伴TP53基因突变的白血病性非淋巴结型套细胞淋巴瘤患者2017年至2023年外周血细胞形态淋巴细胞占比、染色体及NGS结果

日期	成熟淋巴细胞比例（％）^a^	异常单克隆B细胞占比（％）^b^	染色体（CpG+IL-2刺激）	NGS
2017-05-26	NA	30.12	46,XY,t（11;14）（q13;q23）[6]/47,XY,idem,+15[1]/48,XY,+4,+15[1]/46,XY[2]	CCND1基因突变检测阳性（NM_053056.2:c.139T>A:p,C47S）；突变比例为9.32％； TP53基因突变检测阳性（NM_000546.6:c.532C>G:p.H178D）；突变比例为18.16％； KMT2D基因突变检测阳性（NM_003482.3：c.12815G>A；p.G4272D）；突变比例为52.78％。
2019-07-18	78	NA	46,XY,t（11；14）（q13；q23）[3]/47,XY,idem,+15[1]/48,XY,+4,+15[1]/47,XY,+18[1]/46，XY[2]	NA
2023-09-18	67	NA	46,XY,t（11；14）（q13；q23）[2]/48,idem,+4,+15[2]/46,XY[6]	TP53突变基因检测阳性（NM_000546.6:c.532C>G：p.H178D）；突变比例为23.68％； KMT2D基因突变检测阳性（NM_003482.3:c.12815G>A：p.G4272D）；突变比例为49.41％; CCND1基因突变检测阳性；（NM_053056.2:c.139T>A：p.C47S）；突变比例为23.33％。

**注** ^a^外周血涂片；^b^外周血流式细胞术肿瘤细胞表型检查；“NA”表示未检测

## 讨论及文献复习

传统认为MCL是一种治疗效果差、易复发的高度侵袭性淋巴系统肿瘤。但nnMCL患者呈惰性病程，长期无需治疗，3年生存率明显高于其他cMCL患者（92％对69％，*P*＝0.006）[6]。nnMCL患者临床表现多无明显淋巴结肿大，疾病主要累及外周血、骨髓及脾，IGHV有突变、CD5阳性、SOX11多为弱表达或阴性，并可表达CD23和CD200[5]。

本例在初次诊断时被误诊为CLL。nnMCL和CLL均可表现为白血病样的临床表现。形态学方面，该患者的外周血淋巴细胞体积较小，大多数小于2个红细胞大小、细胞大小一致且形态规则、核染色质浓集、中间有白色副染色质、类似于CLL中典型的龟背状淋巴细胞[7]。这种形态特征与典型MCL中常见的中等偏大的细胞体积和较多的幼淋细胞特征不同，属于MCL中较为罕见的小细胞亚型[8]。从免疫表型来看，本例肿瘤细胞CD5和CD23阳性、CD200弱阳性（dim）。Saksena等[9]研究发现，13％（103/798）的MCL患者流式细胞术检测CD23阳性，但与典型CLL中CD23强阳性的表达模式不同，MCL中CD23通常表现为部分阳性或弱阳性。CD23阳性的MCL患者较多伴随CD200阳性、SOX11阴性，并表现为白血病样、非淋巴结型。同样，Hu等[10]报道，4％（25/668）的MCL患者流式细胞术检测CD200阳性，可表现为阳性或部分阳性并常伴随CD23阳性、SOX11阴性。由于CD200和CD23的表达都支持CLL的诊断，此类患者诊断时与CLL鉴别诊断困难。但与典型的CLL不同，CD23、CD200阳性的MCL患者常常表达更高强度的B细胞表面标志物（如CD20、CD22和CD79b）以及表面免疫球蛋白轻链；然而，这与不典型CLL仍有相当大的重叠[11]。该患者流式细胞术CLL免疫表型积分3～4分，易误诊为CLL，提示流式细胞术在B淋巴细胞增殖性疾病的鉴别中仍具有局限性，应当结合骨髓活检、淋巴结活检cyclinD1和SOX11免疫组化及t（11;14）（q12;q23）遗传学检测等结果，综合对CLL和MCL进行鉴别诊断[12]。

本例伴有TP53基因突变，且在长达12年的时间里未出现治疗指征。在MCL患者中，TP53基因突变的发生率较高，达到11％。传统观点认为，携带TP53基因突变的MCL患者属于高风险群体，常伴有如Ki-67指数≥30％、复杂核型、母细胞/多形性形态等高风险因素，这些患者对免疫化疗的反应较差，PFS期和OS期显著缩短[3],[13]–[14]。即便是在新药时代，如WINDOW-1研究中伊布替尼联合利妥昔单抗的A阶段治疗，TP53基因异常的患者完全缓解（CR）率相对较低（55％对91％，*P*＝0.046）[15]。因此，临床上及多项研究中已将TP53基因突变状态列为具有预后价值的重要分子标志，并且对MIPI起到补充作用[16]–[17]。

本例nnMCL患者NGS结果提示存在TP53基因p.H178D突变，该突变位点被证实为体细胞突变，在临床基因组资源中心数据库（ClinGen）和通用突变数据库（UMD）中均提示该突变位点可能致病[18]。尽管如此，患者长期病情稳定，无需治疗。这种情况并非个例。在cMCL和nnMCL患者中，TP53基因异常的出现频率相近（36％对38％）。cMCL的基因组复杂程度高和TP53基因异常预示较短的OS期，但在nnMCL中，仅基因组复杂程度与较短的首次治疗时间（TTFT）和OS相关，TP53基因异常并未显示出明显的相关性[6]。即使在同样存在TP53基因异常的情况下，nnMCL相比于cMCL也有更长的生存期。这一现象与部分处于Binet A期且IGHV基因有突变的CLL患者相似[19]。

一项包含26例MCL患者的研究[20]显示，3例TP53基因突变的nnMCL中有2例为亚克隆（等位基因变异频数≤10％），而cMCL则为主克隆TP53基因突变。Yi等[21]通过基因组学将MCL分为4个亚群，本例符合其中C1群的患者特征：低Ki-67指数、IGHV有突变、单独TP53基因突变且不伴有TP53基因缺失或NOTCH1、CDKN2A等不良基因异常，这部分患者多为记忆B细胞起源，TP53基因突变与野生型患者之间OS差异无统计学意义（*P*＝0.47）。尽管这些观察结果尚需通过前瞻性研究来验证，但它们确实在一定程度上解释了nnMCL与cMCL中TP53基因突变预后差异的现象，同时也表明，TP53基因突变的存在本身不应成为启动治疗的决定性因素。

目前尚无前瞻性研究指导nnMCL的治疗，但大多数回顾性研究数据表明，对于无症状的nnMCL患者应采取观察等待策略，避免过度治疗[22]–[23]，可参考类似于国际慢性淋巴细胞白血病工作组（iwCLL）治疗CLL的指征[24]。但需要指出的是，携带TP53基因突变和（或）缺失的nnMCL仍属于疾病进展的高风险群体。Chapman-Fredricks等[25]报告了3例携带TP53、ATM和（或）13q14缺失的nnMCL患者，患者均出现了疾病进展。Mori等[26]也报道了2例具有TP53基因异常且需要治疗的nnMCL患者，患者在5个月到5年不等的时间内出现治疗指征，通过IR（伊布替尼+利妥昔单抗）方案治疗及自体造血干细胞移植巩固取得了CR。

IMCL-2015（NCT02682641）研究系统探索了IR方案在经基因表达谱鉴定的17例nnMCL患者和14例cMCL患者中的疗效。在中位随访3年时，4例患者出现疾病进展，其中3例为携带TP53基因突变的nnMCL患者、1例为不携带TP53基因突变的cMCL患者。MIPI评分和TP53基因状态被认为是影响PFS的重要因素，而非nnMCL与cMCL的分型，进一步证实TP53基因异常仍是MCL的高危不良预后因素[27]。因此，对于携带TP53基因异常的nnMCL患者，采取更为密切的随访和疾病监测是合理的管理策略。

综上，临床上需要注意区别nnMCL患者，患者临床表现、肿瘤细胞免疫表型及细胞形态学等方面可能与CLL患者接近，完善cyclin D1免疫组织化学检测及t（11;14）的FISH检测可以进行有效鉴别。对于单独的TP53基因突变，当患者存在IGHV基因有突变且不伴有TP53基因缺失，NOTCH1、CDKN2A基因异常等不良预后因素时，疾病进程可能仍然表现为惰性，早期治疗并不能改善这些患者的预后。然而，对于伴有TP53基因异常等高危因素的nnMCL患者仍需要密切随访、监测疾病进展，综合评价患者的一般情况、治疗指征和整体生物遗传学特征后选择治疗方案。
